# Using a Community Based Participatory Research Approach to recruit American Samoans for Alzheimer’s Disease and Related Dementia Research

**DOI:** 10.1002/alz.089855

**Published:** 2025-01-09

**Authors:** Grace Tuatoo

**Affiliations:** ^1^ American Samoa Community, Pago Pago, AS American Samoa

## Abstract

**Background:**

The *Puipui Malu Manatu* (PMM) study (RF1AG075904) is determining Alzheimer’s Disease and Related Dementias (ADRD) prevalence by administering cognitive assessment tools and determining prevalence of known genetic and plasma biomarkers. The sampling method uses a cluster and selection process to obtain a randomized sample of 1089 adults that is generalizable to the population. Research hesitancy exists due to historical abuse of non‐Indigenous researchers conducting studies that are not reflective of the community health needs. Community based participatory research (CBPR) approaches utilize diverse stakeholders in various levels of a study to ensure trust, respect, and reciprocity between the researchers and a community. Our previous studies have shown using CBPR approach with a cultural lens that shows *fa’aaloalo* (respect) have been effective in recruiting participants.

**Methods:**

We incorporated CBPR approaches that follow the *Fa’aSamoa* (Samoan way/culture) to promote awareness of the PMM objectives and recruit eligible participants. The adherence of cultural protocol was accomplished by presenting at the Office of Samoan Affairs (OSA). The OSA is comprised of the *Pulenu’u* (village mayor), led by an *Ali’i* (Paramount Chief), and is the cultural governance body for villages. Essentially making them gatekeepers to eligible participants. The Secretary of Samoan Affairs provided a letter of support, granting the access to the villages via *Pulenu’u*. We implemented the “PMM on Wheels” in the remote village of Aoa in collaboration with the *Pulenu’u*.

**Results:**

The support from the OSA and *Pulenu’u* allowed for the first “PMM on Wheels” to be conducted. The staff recruited 10 eligible participants who would not be able to access the two study site locations. Furthermore, we were able to maintain randomization in our sampling strategy. Participants completed cognitive assessments and provided a blood sample. Future events are scheduled in the beginning of 2024.

**Conclusion:**

Negative reactions towards research studies in Indigenous populations have been supported by historical abuses by non‐Indigenous researchers. Utilizing CBPR with a cultural lens in the American Samoa population has shown rebuild trust and *fa’aaloalo* to better the health outcomes of the community.

